# CNN-Based Interpretable Feature Extraction Methods Considering Pairwise Interactions

**DOI:** 10.3390/s25185634

**Published:** 2025-09-10

**Authors:** Kyuchang Chang, Sujin Lee, Jun-Geol Baek

**Affiliations:** 1 Department of Artificial Intelligence, Jeju National University, Jeju 63243, Republic of Korea; kyuchang@jejunu.ac.kr; 2 LG Electronics, Seoul 07796, Republic of Korea; 3 School of Industrial and Management Engineering, Korea University, Seoul 02841, Republic of Korea

**Keywords:** interaction effect, convolutional neural network (CNN), eXplainable artificial intelligence (XAI), pairwise interaction, feature extraction, multivariate time series classification

## Abstract

This paper proposes a framework that improves classification performance for multivariate time series data while providing an objective assessment of each variable’s influence, including interaction effects. While convolutional neural networks (CNNs) offer significant advantages in analyzing multivariate time series data, the structural limitations of CNNs have restricted their ability to detect statistical interactions. Our approach creatively modifies convolutional filters and layer structures, enabling feature extraction that captures the influence of pairwise interactions. These extracted features are processed by interpretable models to calculate feature importance, enabling in-depth causal analysis by quantifying both individual and pairwise variable effects. In addition, the proposed method enhances the overall classification performance of multivariate time series data. Synthetic data experiments verified that the proposed method effectively extracted relevant features that explain pairwise interactions. In addition, in the multivariate time series classification experiments using real data, the proposed method demonstrated superior performance compared to baseline methods. These results suggest that the proposed approach is a practical and interpretable solution for multivariate time series classification tasks in domains where variable interactions play a decisive role, such as healthcare, finance, and manufacturing.

## 1. Introduction

Many scientific studies focus on identifying significant variables and elucidating their effects. Research harnessing the structural properties of machine learning models for core tasks such as classification and prediction has been a longstanding area of interest. Another important focus has been the use of these models to pinpoint causal variables. The process of identifying causal variables requires an objective quantification of each variable’s influence. However, when some variables interact, their effects cannot be decomposed into independent lower-dimensional parts and must be modeled jointly. The study of interaction detection has a long history in statistics, with its origins tracing back to the 1920s when two-way ANOVA was introduced [1].

The interactions examined in this study refer to statistical interactions, where the effect of one variable on the output is contingent on the values of other variables [2]. In other words, a statistical interaction refers to a phenomenon in which two or more variables combine to affect the results, except for the additive and subtractive effects of variables [3]. When conducting a causal analysis in this study, considering the influence of an interaction refers to measuring it in the same way as the variable importance of individual variables. Statistical interactions provide valuable insights into how features jointly influence an outcome. Identifying these interactions is particularly beneficial for scientific discoveries and hypothesis testing. For instance, physicists might seek to understand which combined factors indicate the presence of new elementary particles, while doctors may want to identify interactions considered in risk prediction models and compare them with known interactions from existing medical research. In terms of manufacturing applications, it is important for engineers to determine which sensor has the greatest impact on the results of the FDC (Fault Diagnosis and Classification) model. Engineers can then identify the cause of an anomaly and take immediate action.

Since the 1920s, various methodologies for interaction detection have been developed. The first category of approaches involves conducting individual tests for each combination of features [4]. Notable approaches like ANOVA and Additive Groves [5,6] fall into the first category. Two-way ANOVA has long been a standard method for detecting pairwise interactions, conducting hypothesis tests for each interaction candidate using F-statistics. Beyond two-way ANOVA, three-way ANOVA performs similar analyses but considers interactions among three variables instead of two. ANOVA is predicated on several assumptions, including normality, homogeneity of variances, and independent observations. If these assumptions are violated, the results may be unreliable. Additionally, ANOVA requires a sufficiently large sample size to produce accurate results. With an insufficient sample size, results may lack statistical significance, making it difficult to draw meaningful conclusions from the data. The second category of methods pre-specifies all potential interaction forms and then employs lasso to select the important ones [7,8]. Lasso-based methods are widely favored due to their efficiency in selecting interactions. An additive model can be constructed with numerous interaction terms, and lasso will shrink the coefficients of insignificant terms to zero [7]. Although lasso methods are fast, they necessitate the specification of all interaction terms of interest. For pairwise interaction detection, this entails (*p*2) terms, and the number increases exponentially for higher-order interaction detection. The third category leverages neural network architectures to detect interactions by interpreting learned model weights. For example, the NIT (Neural Interaction Transparency) approach disentangles shared learning across interactions by using blocked network structures, providing an interpretable framework for analyzing pairwise interactions [3].

Various methodologies have been developed to detect interaction effects in this manner. The proposed methodology distinguishes itself from previous research by utilizing the CNN (convolutional neural network) algorithm to enhance the performance of classification tasks on multivariate time series data while simultaneously detecting quantitative interaction effects. This paper proposes a novel CNN-based interpretable feature extraction framework that not only improves classification performance but also quantifies the effects of individual variables and pairwise interactions in multivariate time series data.

With the development of sensor and data storage technology, multivariate time series data are collected in various industries. Time series data are a collection of quantities assembled over time and ordered chronologically. Multivariate time series data refer to a set of time series data collected from multiple sensors. Multivariate time series classification is aimed at predicting class labels for multivariate time series data and is regarded as an important but challenging problem in data mining [9]. Multivariate time series classification can be applied in various fields, such as manufacturing processes that require real-time anomaly detection and activity recognition research that analyzes motion types. In the past, domain knowledge-based hand-crafted features were extracted from multivariate time series data. Then, multivariate time series classification was performed after analyzing the extracted features by time point or section. However, with the increasing complexity and volume of multivariate time series data, applying the existing feature extraction method is expensive and it is difficult to expect high performance. Recently, several deep learning models that automatically perform both feature extraction and classification tasks have been developed for multivariate time series classification, demonstrating high performance. One notable example is the convolutional neural network (CNN), a type of deep neural network widely used in various fields such as computer vision, natural language processing, and speech recognition due to its ability to directly analyze raw data without extensive preprocessing [10]. A deep convolutional network uses the hierarchical layers of tiled convolution filters to mimic the effects of receptive fields and is known for good performance in the feature extraction of two-dimensional or higher-dimensional matrix data. Therefore, various attempts have been made to use CNNs in various fields other than images, with numerous studies exploring their use in multivariate time series classification [11].

Despite the advantages of CNN models, deep learning models including CNNs are often referred to as black-box models, making it challenging to interpret their results due to their complex internal structures. Recently, eXplainable Artificial Intelligence (XAI) research to describe the purpose, rationale, and decision-making process of deep learning models in a way that can be understood by humans has been actively conducted [12,13]. XAI research dealing with CNN structures mainly focuses on a causal analysis of image data. Meanwhile, certain studies have been extended to multivariate time series data. For instance, one study introduced a receptive field tailored to multivariate sensor signals sliding along the time axis to extract fault features, enabling the identification of variables and time segments representing process faults [14]. One study applied a class activation map (CAM), a widely used method for analyzing causal relationships in image data, to multivariate time series data [15]. A gradient-based method called grad-CAM is implemented to back-propagate gradients relative to a specific class and visualize the attention patterns of the network over a multivariate time series. The importance of each variable is expressed through a heat map. In addition, the model-agnostic method can be used for a causal analysis of multivariate time series data. The model-agnostic approaches extract post hoc explanations by treating the original model as a black box. Canizo et al. (2019) studied the main challenges of these methods and reviewed a model-agnostic explanation approach (LIME) [16] that addresses these challenges. The model-agnostic method is also applicable to CNN models and can determine the cause of multivariate time series data. Existing CNN-based methodologies perform causal analysis by considering only the influence of individual variables making it difficult to analyze the effects of complex interactions involving two or more variables.

In this study, interactions were explicitly considered when performing a causal analysis of multivariate time series data. This study proposes a novel CNN-based interpretable feature extractor for analyzing multivariate time series classification data to extract features to quantify the effects of both individual variables and pairwise interactions. The proposed model, Interaction CNN (InCNN), has the same number of parallel convolution layers as the number of input data variables. The parallel convolution layers include separate layers for extracting individual variable features and pairwise interaction features. Through a flattening process, the final feature maps are transformed into a feature vector that captures the influence of individual variables and pairwise interactions. By inputting the generated feature vector into interpretable models, the influence of individual variables and their pairwise interactions can be analyzed effectively. The application of the proposed method is simple in that existing classifiers such as random forest or logistic regression can be utilized as they are. The advantages of the proposed method are as follows:

- As the InCNN extracts features that can explain the influence of not only individual variables but also pairwise interactions, it helps users to trace the causative factor, including the interaction effect.
- Because the InCNN is based on a high-potential CNN structure, it can be applied across various fields where multivariate time series data are generated.
- The InCNN is user-friendly, as causal variables can be inferred by simply inputting the flattened feature vector into models that provide variable importance.

Experiments were conducted using two datasets to evaluate the performance of the proposed method. First, simulation data were used to test whether the InCNN pairwise interaction features uncovered major causal factors. In addition, we compared the classification performance of the classifier using InCNN feature vectors with existing methodologies for multivariate time series classification on actual multivariate time series data.

The remainder of this paper is organized as follows. Section 2 introduces the proposed method in detail. Section 3 describes the experimental results to demonstrate the efficiency of the proposed methodology. Section 4 provides the discussion and conclusions, along with recommendations for future research.

## 2. Materials and Methods

In this section, the entire structure and modeling method of the InCNN are described. First, a description of the multivariate time series classification dataset and the data preprocessing method used to train the CNN models is provided. Then, the convolution process in the convolution layer is explained to clarify the feature extraction process in the InCNN. The entire model structure and components of the InCNN are covered. The training process for the InCNN is then described. Finally, the method of inferring causal variables using feature vectors extracted from the InCNN and the methodology of calculating variable importance used in this study are presented.

### 2.1. Data Preprocessing

Multivariate time series data refer to time series data collected from multiple sensors in chronological order. We assumed that the length of the time series data is *T* and the number of sensors is *v*. Therefore, the univariate time series data of variable *i* is given by $x_{i}=\left[x_{i1},x_{i2},\ldots ,x_{iT}\right]$.

The data for the *v* variables are given by $X=[x_{1},x_{2},\ldots ,x_{v}]$.

Multivariate time series classification data refer to data in which class *y* is assigned to multivariate time series data X.

If *N* multivariate time series classification data are collected, the dataset is given by $D=\{\left(X_{1},y_{1}\right),\left(X_{2},y_{2}\right),\ldots ,\left(X_{N},y_{N}\right)\}$.

The multivariate time series classification data X become a two-dimensional matrix when the number of collected data is two or more. This two-dimensional matrix X can be directly input into the CNN. However, training a CNN model requires a large amount of data. If the number of available multivariate time series instances is small, it becomes difficult to train the CNN model. In addition, when *T* is very long, the high-level features extracted using the convolution layers have difficulty reflecting the features of local temporal parts in the data. If there is a possibility that the class can be changed over time in the test data, the training data must be segmented regionally. By training the CNN model with regionally segmented data, the model can learn local features in the training data, and local parts of the test data can be classified. The sliding window is a data preprocessing method that can be applied when the amount of data is small or a local analysis of the data is required [17]. When analyzing time series data using CNN in various studies, sliding windows are used as a preprocessing method [18,19,20]. To use the sliding window, the user must define its length and moving interval. If the length of the sliding window is set to *t*, and the moving interval is set to *s*, the sliding window moves *s* steps along the time axis, creating new features containing *t* multivariate data. For example, assuming that a sliding window is applied to univariate time series data, the first data regenerated by the sliding window with length *t* and interval *s* is univariate time series data $x_{i}=\left[x_{i1},x_{i2},\ldots ,x_{it}\right]$. After applying the sliding window, the *v* univariate data come together to form $X_{1}^{\prime }$, which is denoted as $X_{1}^{\prime }=\left[x_{1},x_{2},\ldots ,x_{v}\right]$. Subsequently, the sliding window generates the second feature of data $x_{i}=\left[x_{\left(i1+s\right)},x_{\left(i2+s\right)},\ldots ,x_{\left(it+s\right)}\right]$ at the position moved by *s* on the time axis. Similarly, *v* features are collected, and the second feature set $X_{2}^{\prime }=\left[x_{1},x_{2},\ldots ,x_{v}\right]$ is created. Thus, the sliding window slices the original multivariate time series data into several local feature sets by moving user-defined steps along the time axis. The total number of data points obtained through the sliding window is (T−t)/s+1. The label of each of the (T−t)/s+1 features is assigned based on the original data.

### 2.2. Convolution Process

The convolution layer in the CNN model extracts features from the input data. In the convolution layer, a convolution operation is performed between the input data and the convolution filter. The number and size of convolution layers and filters are typically specified by the user based on the size and complexity of the data. For noisy or high-dimensional data, multiple deep convolution layers help extract meaningful features. When data pass through a convolution layer, summarized features containing essential information are extracted. Data that have passed through several convolution layers are transformed into high-level features that are robust to noise while maximizing class distinction.

When the data are inserted into the convolution layer, the convolution filter performs a convolution operation between the data and the weights in the filter to generate a feature map. Suppose that the input data have height *h*, width *w*, and channels *c*. For clarity, the size of the 3D matrix is indicated in the order of channel, height, and width in the form (c,h,w). Here, *c* denotes the number of channels corresponding to the variables or sensors in the multivariate time-series data, while *h* and *w* represent the temporal length and the pseudo-spatial dimension derived from preprocessing.

This definition is provided to ensure that readers can unambiguously interpret the notation used throughout the paper. Assuming that there is a convolution filter with the same height and width *k* in the convolution layer, the size of one convolution filter is (c,k,k). Then, the size of the feature map generated by the convolution operation is:

$$(1,\frac{h+2p-k}{s}+1,\frac{w+2p-k}{s}+1),$$

where *p* is the size of the padding and *s* is the stride of the filter.

The convolution layer has a user-specified number of convolution filters. When there are *n* convolution filters, each convolution filter performs an operation on the input data, and the final feature map is created in the shape of:

$$(n,\frac{h+2p-k}{s}+1,\frac{w+2p-k}{s}+1).$$

### 2.3. Model Structure

**Figure 1:**
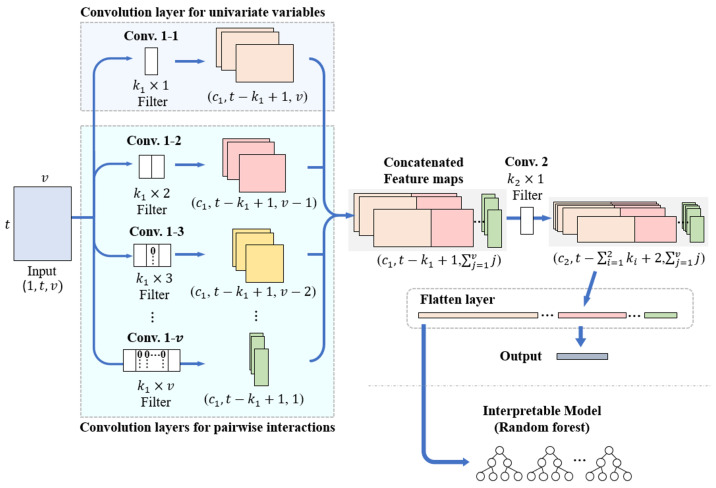
InCNN architecture.

The InCNN proposed in this paper serves as a feature extractor, capturing features of individual variables and pairwise interactions from multivariate time series classification data. The InCNN consists of parallel convolutional layers designed to extract features of individual variables and pairwise interactions, followed by subsequent convolutional layers and fully connected layers. The subsequent convolutional layers are responsible for extracting high-level features. Figure 1 illustrates the structure of the InCNN. For the convenience of explanation, the structure is described as comprising the parallel convolution part, which is Conv. 1–*i*(i=1,2,…,v), and a single subsequent convolution layer, which is Conv. 2.

After preprocessing with a sliding window, the original data were converted into data with *v* variables and *t* time points. The input data were a matrix of size (1,t,v). The input data were duplicated and entered into *v* convolution layers in the parallel convolution part. The parallel convolution part is shown in Figure 1, and each convolution layer in parallel is denoted as Conv. 1–*i*.

The convolution filters in the parallel convolution part had the same height and number of channels and differed only in the width of the filter. In Figure 1, the heights of the convolution filters of Conv. 1–*i* and Conv. 2 are expressed as $k_{1}$ and $k_{2}$, and the number of channels as $c_{1}$ and $c_{2}$. Similar to a general CNN model, the height, width, and number of channels of the convolution filters were determined as hyperparameters and could be specified differently by the user according to the complexity and size of the input data. However, the same height and number of channels had to be applied to the convolution filters in Conv. 1–*i*. The stride of all convolution layers in the InCNN was set to 1, and the padding size was set to 0.

In the parallel convolution part of InCNN, Conv. 1–1 is a convolution layer used to extract the features of individual variables. Conv. 1–2 through Conv. 1–*v* are convolution layers for extracting pairwise interaction features between the two variables.

The input data are transformed into a feature map through this parallel convolution layer part. Each column of the feature map resulting from Conv. 1–1 contains features of individual variables, whereas each column of the feature map resulting from Conv. 1–2 to Conv. 1–*v* contains the pairwise interaction features between the two variables. Because the height of each feature map is the same as $k_{1}$ and the convolution filters in Conv. 1–*i* have the same height and same number of channels, it is possible to form an integrated feature map by concatenating the columns of all feature maps. Conv. 2, the subsequent convolution layer, merges the feature maps from the parallel convolution layers to obtain higher-level, abstract features. Conv. 2 uses convolution filters with heights of $k_{2}$ and widths of 1. The width of the convolution filters in Conv. 2 is set to 1 to extract the high-level features of each column without mixing features contained in each column of the integrated feature map. More subsequent convolution layers could be used depending on the complexity of the data. However, the width of the convolution filters in the subsequent convolution layers is 1 for the same reason.

The final feature map, which is an output of the subsequent convolution layer, undergoes a flattening process in the flatten layer that is connected to the output layer without any hidden layers. When the training process is complete, the InCNN flattened feature map is converted into a vector containing the information of individual variables and pairwise interactions in order. The influence of individual variables and pairwise interactions could be quantified by inputting the feature vector into classifiers that can extract variable importance. In this study, several experiments were conducted using a random forest to extract the variable importance.

#### 2.3.1. Parallel Convolution Layers

**Figure 2:**
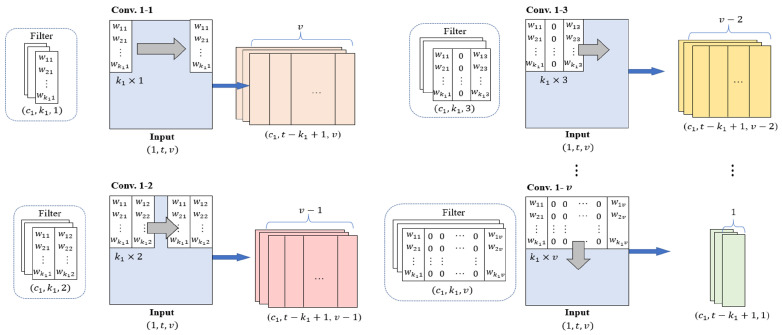
Parallel convolution layers in InCNN.

InCNN includes convolution layers in parallel, where the number of layers is the same as the number of variables in the input data. The convolution filters in each convolution layer extract features of individual variables and pairwise interactions between variables. Figure 2 illustrates the design of convolution filters and the feature map generated through the convolution operation.

Conv. 1–1 is a convolution layer for the feature extraction of individual variables, and Conv. 1–2, Conv. 1–3, …, Conv. 1–*v* are the convolution layers for the feature extraction of pairwise interactions between two variables. The weight of the *i*th row and the *j*th column in the filter is expressed as $w_{ij}$ in Figure 2.

In Conv. 1–1, the convolution filter of size $k_{1}\times 1$ moves to the right by one stride to extract the features of the individual variables. The first column of the output feature maps from Conv. 1–1 contains the features of the first variable.

In Conv. 1–2, the convolution filter of size $k_{1}\times 2$ moves to the right by one stride and extracts the pairwise interaction features between two variables located next to each other. Therefore, the first column of the feature maps from Conv. 1–2 contains the pairwise interaction features of the first and second variables. The number of columns of feature maps from Conv. 1-2 is v−1.

The convolution filters in each convolution layer from Conv. 1–3 to Conv. 1–*v* are filled with zeros, except for the first and last columns. A convolution filter of size $k_{1}\times 3$ in Conv. 1-3 extracts the pairwise interaction features between two variables located one column away. The first column of the output feature maps from Conv. 1–3 contains the pairwise interaction features of the first and third variables. The number of columns of the feature maps from Conv. 1–3 is *v*–2.

This approach is repeated across all variable pairs, with Conv. 1–*v* using the $k_{1}\times v$ filter to extract pairwise interaction features. The number of columns of the feature maps passed through Conv. 1–*v* is 1. These feature maps contain the pairwise interaction features of the first and last (*v*th) variables.

Therefore, the feature maps generated through all parallel convolution layers have the same height and number of channels. The feature maps of Conv. 1–1 contain the features of individual variables in each column, whereas the feature maps of Conv. 1–2 through Conv. 1–*v* contain the pairwise interaction features in each column.

#### 2.3.2. Flattening Process

Each column of the feature map that is generated through all the convolution layers in InCNN contains the features of individual variables and pairwise interactions between two variables. The size of the generated feature map at the end is $\left(c_{2},\;t-\sum_{i=1}^{2}k_{i}+2,\;\sum_{i=1}^{v}i\right)$.

The first column of every feature map contains the features of the first variable, and the last column contains the pairwise interaction features of the first and last variables.

**Figure 3:**
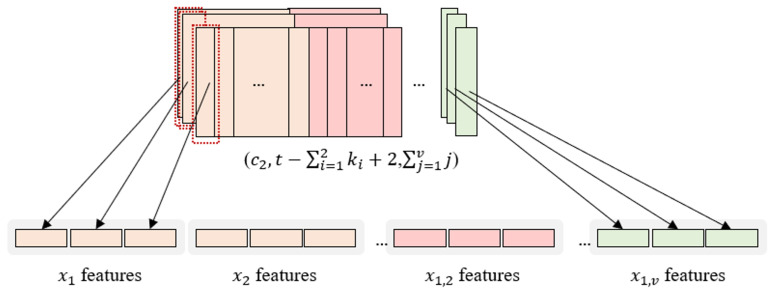
Flattening process.

The flattening process of the InCNN is illustrated in Figure 3. In Figure 3, the individual variable is expressed as $x_{i}$, and the pairwise interaction between the two variables is expressed as $x_{i,j}$ (i≠j; i,j=1,2,…,v).

First, the first column of all feature maps is flattened. Then, the second column of all the feature maps is flattened. Proceeding in this manner, the last row of all feature maps is flattened.

Because the heights and channels of the feature maps are all equal to $t-\sum_{i=1}^{2}k_{i}+2$ and $c_{2}$, respectively, the features of individual variables and pairwise interactions in the flattened feature map are vectors of the same length, that is, $\left(t-\sum_{i=1}^{2}k_{i}+2\right)\times c_{2}$.

Because it is flattened in the order of the variables, it is easy to track the nodes that belong to each variable.

The total number of nodes constituting the flattened feature vector is $\left(t-\sum_{i=1}^{2}k_{i}+2\right)\times c_{2}\times \sum_{i=1}^{v}i$. This is because there are $c_{2}$ feature maps with height $t-\sum_{i=1}^{2}k_{i}+2$, and there are $\sum_{i=1}^{v}i$ number of interaction variables and pairwise interactions.

This flattened feature vector is directly connected to the resulting layer without hidden layers. The hidden layers are not included in the fully connected layer. In other words, we tried to reflect the influence of individual variables and pairwise interactions as much as possible on the feature map by extracting features using only the convolution layers.

### 2.4. Model Training

**Figure 4:**
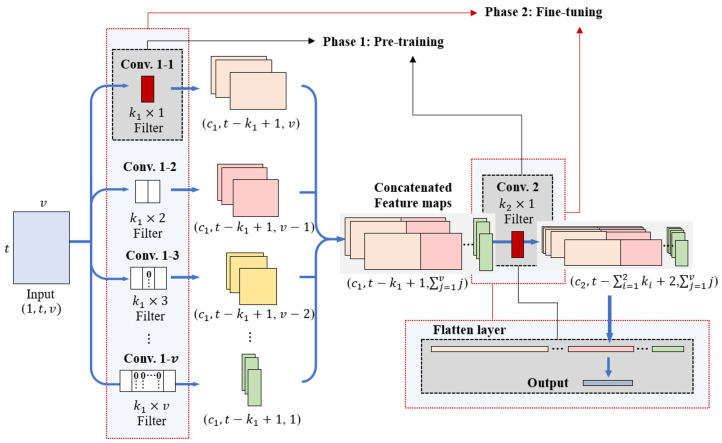
Training process in InCNN.

The training process of InCNN consists of two phases. In phase 1, Conv. 1–1 is used to extract the features of individual variables, and the subsequent convolution layer is used for high-level feature extraction. The parameters of the fully connected layer are pre-trained first. Subsequently, in phase 2, the entire model is fine-tuned. This process is illustrated in Figure 4. This two-phase training process considers the hierarchical ordering principle of the interaction. According to the hierarchical ordering principle, lower-order interactions have a higher priority than high-order interactions in which multiple variables are combined [21]. In addition, high-order interactions exist when there are corresponding low-order interactions [5]. Therefore, even if the results show that high-order interactions are significant whereas low-order interactions, which are components of the high-order interactions, are insignificant when analyzing the causes of data, the interpretation of low-order interactions should be included in causal analysis.

From a training perspective, pre-training on individual variables in phase 1 enables the model to form stable low-level representations while reducing the effect of noise and narrowing the parameter search space. This stabilization allows the subsequent fine-tuning phase to focus on learning higher-order dependencies without disrupting the already learned low-order structures. As a result, the optimization process becomes more stable and converges more efficiently, improving both performance and interpretability.

Learning the features of individual variables through pre-training implies that the influence of individual variables is considered first in forming decision boundaries. Subsequently, the pairwise interaction features of the two variables are learned with fine-tuning. Pairwise interaction features are higher-order interactions than individual variables. Because the features of individual variables are learned prior to learning pairwise interaction features, notably, this training method considers the hierarchical ordering principle in forming decision boundaries.

When conducting experiments using simulation data, it was confirmed that the accuracy of causal variable inference was higher when the two-phase training method was applied than when training without pre-training. These experimental results are consistent with the theoretical rationale, demonstrating that the staged approach effectively improves the stability of learning and the accuracy of causal variable inference. Therefore, InCNN employs a two-phase training method that pre-trains the features of individual variables first and then fine-tunes the entire model.

### 2.5. Causal Analysis

Variable importance refers to techniques that assign scores to input variables based on their usefulness in classifying the given data. By inputting the InCNN feature vector into a classifier that is capable of deriving variable importance, multivariate time series classification data can be classified, and variable importance can be obtained. In this study, the InCNN feature vector was input into a random forest to classify data and derive variable importance. The number of variable importance scores obtained was the same as the number of nodes in the feature vector. The feature vector of individual variables and pairwise interaction terms composed of $\left(t-\sum_{i=1}^{2}k_{i}+2\right)\times c_{2}\times \sum_{i=1}^{v}i$ nodes that were located in order. Therefore, it was straightforward to identify the nodes at specific locations that belonged to each variable. Variable importance can be derived by summing the importance scores of the nodes belonging to each variable. In this study, the mean decrease in impurity (MDI) [22] was used as a metric to measure the variable importance in a random forest. Impurity is an indicator of how mixed data from different classes are in the node of the decision tree. If the data in the node is composed of the same class, the impurity is minimized, whereas when multiple classes of data exist with the same size in the node, the impurity is maximized. The decrease in impurity results from the difference between the impurity of the sum of child nodes and the impurity of the parent node when splitting a node based on a specific variable. If the amount of impurity reduction is large on the specific variable used in dividing nodes, then the variable is considered significant for causal analysis. In one decision tree, the total impurity reduction for each variable can be calculated. The average value of impurity reduction across all decision trees in the random forest constitutes the MDI. The MDI is a representative indicator used as variable importance in random forests.

## 3. Experiments and Results

In this study, we verified whether the extracted feature vector of the InCNN reflected the main cause of the data using simulation data. In addition, the classification performance of the classifier using the InCNN feature vector was confirmed using the actual multivariate time series classification data. The first experiment examined whether the convolution layers in the parallel convolution part generated feature maps that effectively captured real pairwise interactions in the data. For the experiment, simulation datasets with one pairwise interaction as the main cause were generated. Subsequently, the feature vector extracted from the trained InCNN was inserted into the random forest, and when the variable importance was obtained, it was checked whether the pairwise interaction with high variable importance matched the actual main causal pairwise interaction in the data.

The second experiment compared the classification accuracy between the classifier using InCNN feature vectors and the methodologies used for multivariate time series classification using the actual multivariate time series data. In the experiment, the classification accuracy of the classifier using the InCNN feature vector was compared to that of existing CNN models. Additionally, classification performances were compared when the InCNN feature vector and hand-crafted features were input into classifiers to account for the influence of pairwise interaction terms.

The main causal factor in the classification of the generated dataset was a single pairwise interaction in the form of a product of two variables. As datasets were generated subject to the rule in Table 1, it was possible to validate the relationship between the data and the classes. If data generated using complex formulas with multiple variables are transformed into matrix data for classification purposes, the relationship between the generated data and the class may become ambiguous during the transformation process. Therefore, to maintain a clear relationship between the dependent variable and the class label, a relatively simple formula was used, as presented in Table 1, to generate simulation datasets. The generated ten datasets each contained approximately 4700 data points with a shape of 5×5. The experiment was conducted by dividing the training and test data by 70/30. Each class contained approximately 9400 data points.

### 3.1. Detecting Pairwise Interaction Experiment

#### 3.1.1. Data Description

For this experiment, simulation data were generated in a two-dimensional matrix format suitable for InCNN learning. First, five independent variables with a uniform distribution between −1 and 1 were generated. Then, two of the five variables were selected and multiplied to create a dependent variable. The method of generating simulation data with a uniform distribution and multiplying two variables to generate a pairwise interaction variable was referred to from [8]. The dependent variables of the dataset generated so far were the real values. To convert the continuous dependent variables into class labels for classification, first, min–max normalization was applied to the dependent variable, and then normalized values were divided into five classes: 0–0.2, 0.2–0.4, 0.4–0.6, 0.6–0.8, and 0.8–1.0. Thus, a multivariate dataset for classification was generated, in which one class label corresponded to one data vector. To transform the data into a matrix form, a matrix of size 5×5 was created by randomly extracting five multivariate data belonging to the same class. Thus, data in which one class label was assigned to one matrix data of size 5×5 was finally generated. A total of 10 experimental cases and 10 datasets were constructed by generating class labels with combinations of all possible two variables out of five. The formulas used to generate the class labels of each dataset are listed in Table 1.

#### 3.1.2. Experimental Settings

The simulation data had a clear relationship between class labels and significant variables, and the size of each data was 5×5. InCNN, comprising a parallel convolution part, was used without any subsequent convolution layers for high-level feature extraction. The height of the convolution filter was set to three, and the channel size was set to three. The experiment was conducted 10 times for each dataset. Except for datasets 5, 8, and 10, the number of epochs for pre-training and fine-tuning of InCNN was set to approximately 40–50, the learning rate was 0.01, and the optimizer used was Adam. For datasets 5, 8, and 10, the average number of epochs during pre-training was 9, 36, and 13, respectively. Because the optimal epoch was different for each dataset, the number of epochs required to optimize the model differed accordingly. To confirm the classification performance and analyze the cause, the feature vector of the training data was extracted using InCNN and input into a random forest for training. Subsequently, the feature vector of the test data was extracted using InCNN and inputted into the random forest to measure classification accuracy and to obtain the variable importance of individual variables and pairwise interactions. The random forest model used in the experiment included 100 trees with a maximum depth of 10.

#### 3.1.3. Experimental Results

Table 2 presents the results of variable importance for 10 different experiments. “#” indicates the dataset number, and “T” indicates the target, which is the main cause variable in the dataset #. The “Univariate” column provides the variable importance scores of five individual variables. The “Pairwise interaction” column provides the variable importance scores of the pairwise interactions for all 10 possible combinations. The “Acc” column provides the classification accuracy of test data using the random forest trained with the InCNN feature vector. Variable importance and accuracy were averaged over 10 experiments for each dataset. As observed from the “Pairwise interaction” column in Table 2, the variable importance score of the pairwise interaction that is the cause of datasets was larger than the variable importance score of other pairwise interactions. In all cases, the most important pairwise interaction identified by the InCNN feature vector matched the actual causal interaction in the dataset. The InCNN determined the main cause, which is a pairwise interaction obtained by the multiplication of two variables, irrespective of the location of the variable in the input data. It was natural to interpret that the features of the main pairwise interaction were efficiently reflected in the feature vector from InCNN; therefore, the importance score of the main causal pairwise interaction was high. In addition, compared to the importance score of single variables, the importance scores of each pair of variables that were components of the main causal pairwise interaction were significantly higher. Thus, it was confirmed that the convolution filter of InCNN was effective in detecting important individual variables and pairwise interactions, which are the main causes in data.

### 3.2. Classification Performance Comparison Experiments

#### 3.2.1. Data Description

The activity recognition system based on the multisensory data fusion (AReM) dataset is a multivariate time series classification dataset [23] for human activity recognition and is open to the UCI repository site [24]. The AReM dataset is used to analyze the collected sensor data and classify the type of behavior a person is currently doing. Data were collected using the following process. The received signal strength (RSS) data were generated from a wireless sensor network (WSN) between three sensors attached to a person’s chest and both ankles. Five RSS data were sampled at 20 Hz intervals for 250 ms, and the average and standard deviation of the five samples were used as variables. The dataset included six variables because there were three WSN sensors. Data in the AReM dataset are multivariate time series data in which six variables are collected at 250 ms intervals for 120 s. In other words, the data included 480 time sequences. There are seven classes in the AReM dataset: bending1, bending2, cycling, lying, sitting, standing, and walking. Each class, except bending1 and bending2, include 15 multivariate time series classification data. Bending1 and bending2 contain seven and six multivariate time series data, respectively. In this experiment, six data points per class were used to balance the dataset. If the entire 480 sequence is used as one data point, there are six data points for each class, making it difficult to train the InCNN owing to the small amount of data. Therefore, to increase the amount of data used for training InCNN and other CNN models, the sliding window method was used as a preprocessing technique. By setting the sliding window with a time length of 10 and a moving step of 5, 95 multivariate time series classification data of size 10×6 were generated from one dataset of 480×6. Because a sliding window was applied to all six datasets, the number of data for each class was reconstructed to 570, and because there were seven classes, the total number of data was 3990. The data were split into training, validation, and test sets with a 60/20/20 ratio, yielding 2394/798/798 data points, respectively.

#### 3.2.2. Experimental Settings

Before proceeding with the classification performance evaluation of the classifier with InCNN features, an experiment was conducted to determine whether there were detectable pairwise interactions using the InCNN model in the AReM dataset. To check the existence of pairwise interactions that are detectable by InCNN in the dataset, the InCNN and CNN models without convolution filters for extracting pairwise interactions were compared. The two models share the same structure, except that InCNN includes parallel convolution layers to capture both individual variables and pairwise interaction features. InCNN parallel convolution filters extract the features of individual variables and pairwise interaction features in which two variables are combined, and the fully connected layer of InCNN includes no hidden layer. Therefore, the phenomenon in which the extracted features are mixed with each other in the hidden nodes does not occur in the structure of InCNN. A CNN model that does not use convolution filters for pairwise interactions includes convolution filters with a column size of 1. In other words, the model only has Conv. 1-1 in the parallel convolution part of InCNN. An increase in classification accuracy with InCNN compared to the CNN model without pairwise interaction filters would indicate that pairwise interaction features enhance classification performance. After confirming that there were pairwise interactions in the dataset, a performance comparison experiment was conducted. In this experiment, classification accuracies were compared between random forest and logistic regression models that were trained with the InCNN feature vector and two other CNN models used in multivariate time series classification. One CNN model used as a comparison group was a CNN model that did not use the convolution filters for pairwise interactions, which was used in the previous experiment to check the existence of pairwise interactions in the data. The other CNN model was a 1D convolutional CNN model. The 1D convolutional CNN model is a structure that is often used in multivariate time series classification. The convolution filter has as many columns as the number of variables; therefore, the filter moves in one direction to extract the features of the data. Detailed information on the CNN models used in the experiments is presented in Table 3. The sizes of filters, feature maps of each model, the number of channels, nodes in the flattening layer, and the resulting layer are indicated.

In the pre-training stage of InCNN, the number of epochs was set to 50. In the fine-tuning stage, the number of epochs was set to 550. For all other CNN models in the comparison group, the epoch count was set to 600, and the experiment was performed 10 times. The learning rate was set to 0.01, and the optimizer used was Adam.

After a comparison experiment with CNN models, an experiment was conducted to compare classification accuracy using InCNN feature vectors and hand-crafted features commonly employed to detect pairwise interactions. Data classification performance was compared when InCNN feature vectors and hand-crafted features were inputted into random forest and logistic regression models, respectively. Hand-crafted features were generated by multiplying pairs of variables among the five, considering all possible combinations. The hand-crafted features were created using multivariate data at each time point in multivariate time series data. The experiment was conducted 10 times, and the random forest model used in the experiments included 100 trees at 20 depths.

#### 3.2.3. Experimental Results

First, an experiment was conducted to check whether pairwise interactions were detectable by InCNN in the AReM dataset. Table 4 presents the classification performance of the InCNN and CNN models without convolution filters for pairwise interactions. Using the InCNN model resulted in an approximately 2% improvement in accuracy and F1 score. Therefore, it indicated that there were pairwise interactions that were detectable by the convolution filters of InCNN in the AReM dataset.

After confirming that there were pairwise interactions in the dataset, an experiment was conducted to compare the classification performance of the methods using the InCNN feature vector with other CNN models.

Table 5 summarizes the classification performance of random forest and logistic regression models that use the InCNN feature vector as input, along with the performance of other CNN models. Table 5 provides the classification accuracy for each class, as well as the overall classification accuracy and F1 score for each model. When the random forest algorithm was applied to the InCNN feature vector, the overall accuracy was the highest at 0.902. In the dataset used for this experiment, the method of applying random forest to the InCNN feature vector outperformed the other models. The methodology using InCNN also demonstrated greater applicability than other CNN models due to its superior classification performance. In addition, users could infer causal variables using a classifier that offers variable importance.

Table 6 shows the classification accuracy when the InCNN feature vector was inputted into the random forest and logistic regression and when the hand-crafted features were inputted into the random forest and logistic regression. The random forest models in this experiment were configured with the same depth and number of trees. Both random forest and logistic regression models showed better classification performance when using InCNN as a feature extractor than when using hand-crafted features considering pairwise interactions. Using the InCNN feature vector instead of hand-crafted features for input not only enhances causal analysis involving pairwise interactions but also maintains high classification performance depending on the dataset.

In summary, in the AReM dataset used for the experiments, the method using the InCNN feature vector as input data on the random forest exhibited better classification performance than other CNN models and the method using hand-crafted features. The method combining InCNN with models that provide variable importance demonstrated its usefulness by exhibiting good performance in terms of accuracy and helping users to analyze the main cause in the dataset considering pairwise interactions.

## 4. Discussion and Conclusions

In this study, we propose a novel framework that, given multivariate time series data, maximizes classification performance while objectively assessing the influence of each variable. This method allows for analyzing the influence of individual variables as well as pairwise interactions. The Interaction-CNN (InCNN) proposed in this study is implemented in four steps: data preprocessing using the sliding window technique, training a convolutional model with a newly designed filter to extract features that explain interactions between variables, extracting features using the trained model, and applying an existing interpretable model, such as a random forest, to the extracted features.

In experiments using simulation data, it was confirmed that meaningful features explaining pairwise interactions could be efficiently extracted, and the method demonstrated superior performance compared to existing models in classification tasks involving multivariate time series data.

The proposed InCNN offers several advantages. First, by overcoming the structural limitations of traditional CNN models, it can extract features that explain the influence of individual variables as well as pairwise interactions. This capability is significant because it allows for a deeper analysis by considering interactions between variables rather than focusing solely on individual variables in causal analysis. Second, the proposed algorithm adapts high-potential CNN model structures for multivariate data, suggesting that InCNN could be applied across a wide range of multivariate datasets. Finally, the proposed method is convenient to use, as it can be combined with various validated white-box methodologies for interpretation. For example, it can be integrated with a random forest model to obtain quantified variable importance, allowing for the choice of a variable importance calculation method depending on the context.

Moreover, by modifying the CNN model to extract pairwise interaction features, it is possible to design filters for third-order and higher-order interactions. While the conceptual approach for extending to three variables follows the same underlying principle as for two variables, its practical realization presents specific challenges. These include adapting the model training methodology to effectively capture more complex interaction structures and designing rigorous validation procedures to confirm that the extracted higher-order interaction features are both meaningful and reliable. Addressing these aspects will require additional investigation and targeted experimentation. Therefore, further research will focus on refining the learning process for higher-order interactions and validating these methods on diverse datasets. Due to the lack of real data to verify the causal relationship between classification results and variable interactions, additional validation using real data will be necessary in future studies.

Beyond the tested simulation setting, the InCNN framework holds strong potential for broader application domains such as finance and healthcare, where identifying and interpreting variable interactions is critical. To enable the empirical validation of interaction effects in datasets characterized by meaningful cross-variable relationships, the proposed method will be applied to real-world data in collaboration with domain experts. Such studies will not only strengthen the interpretability of the proposed framework but also demonstrate its value across diverse multivariate time-series analysis scenarios.

## Figures and Tables

**Table 1:** Mathematical expressions for generating main cause in dataset.

Data Num	Expression	Data Num	Expression
Data1	$x_{1}\times x_{2}$	Data 6	$x_{2}\times x_{4}$
Data2	$x_{2}\times x_{3}$	Data 7	$x_{3}\times x_{5}$
Data3	$x_{3}\times x_{4}$	Data 8	$x_{1}\times x_{4}$
Data4	$x_{4}\times x_{5}$	Data 9	$x_{2}\times x_{5}$
Data5	$x_{1}\times x_{3}$	Data 10	$x_{1}\times x_{5}$

**Table 2:** Importance scores of univariate variables and pairwise interactions using features extracted by InCNN.

		Univariate Variables					Pairwise Interactions										Acc
#	T	$x_{1}$	$x_{2}$	$x_{3}$	$x_{4}$	$x_{5}$	$x_{1,2}$	$x_{2,3}$	$x_{3,4}$	$x_{4,5}$	$x_{1,3}$	$x_{2,4}$	$x_{3,5}$	$x_{1,4}$	$x_{2,5}$	$x_{1,5}$	
1	$x_{1,2}$	**0.20** **7**	**0.20** **5**	0.00 2	0.00 1	0.00 1	**0.39** **1**	0.03 7	0.00 2	0.00 2	0.03 6	0.03 2	0.00 1	0.04 1	0.04 1	0.00 0	0.99 7
2	$x_{2,3}$	0.00 2	**0.21** **3**	**0.21** **6**	0.00 2	0.00 1	0.02 2	**0.42** **8**	0.02 9	0.00 2	0.00 1	0.03 4	0.03 7	0.00 0	0.01 3	0.00 0	0.99 6
3	$x_{3,4}$	0.00 1	0.00 2	**0.16** **8**	**0.15** **7**	0.00 2	0.00 2	0.03 7	**0.55** **6**	0.04 4	0.01 2	0.01 2	0.00 6	0.00 0	0.00 0	0.00 0	0.99 2
4	$x_{4,5}$	0.00 1	0.00 1	0.00 2	**0.20** **6**	**0.20** **0**	0.00 2	0.00 2	0.03 4	**0.46** **3**	0.00 1	0.02 0	0.02 0	0.01 9	0.01 6	0.01 2	0.99 4
5	$x_{1,3}$	**0.15** **0**	0.00 1	**0.19** **3**	0.00 1	0.00 2	0.07 5	0.00 4	0.10 1	0.00 2	**0.30** **3**	0.00 1	0.04 0	0.03 5	0.00 0	0.09 0	0.99 6
6	$x_{2,4}$	0.00 2	**0.23** **4**	0.00 2	**0.22** **8**	0.00 2	0.07 7	0.02 4	0.07 3	0.02 3	0.00 2	**0.31** **1**	0.00 1	0.01 2	0.01 0	0.00 0	0.99 6
7	$x_{3,5}$	0.00 2	0.00 1	**0.23** **5**	0.00 2	**0.24** **4**	0.00 2	0.04 9	0.03 4	0.05 3	0.02 7	0.00 1	**0.34** **8**	0.00 0	0.00 0	0.00 1	0.99 7
8	$x_{1,4}$	**0.19** **3**	0.00 2	0.00 2	**0.20** **2**	0.00 2	0.08 0	0.00 3	0.08 8	0.09 1	0.05 4	0.00 4	0.00 1	**0.21** **5**	0.00 1	0.06 0	0.99 8
9	$x_{2,5}$	0.00 3	**0.28** **6**	0.00 3	0.00 3	**0.28** **9**	0.01 7	0.01 0	0.00 2	0.01 9	0.00 0	0.00 0	0.00 0	0.00 2	**0.36** **7**	0.00 0	0.99 5
10	$x_{1,5}$	**0.24** **8**	0.00 2	0.00 2	0.00 2	**0.26** **8**	0.09 4	0.00 4	0.00 4	0.05 0	0.05 1	0.00 2	0.00 7	0.02 0	0.06 5	**0.18** **1**	0.99 6

**Table 3:** Summary of configurations of CNN models used in AReM experiments.

	InCNN			CNN Without Interaction Filters			1D CNN		
	Filter	Ch	Output	Filter	Ch	Output	Filter	Ch	Output
Convolution layer 1	(5,1),…,(5,6)	3	(3,6,21)	(5,1)	3	(3,6,6)	(5,6)	3	(3,6,1)
Convolution layer 2	(3,1)	5	(5,4,21)	(3,1)	5	(5,4,6)	(3,1)	5	(5,4,1)
Convolution layer 3	(3,1)	7	(7,2,21)	(3,1)	7	(7,2,6)	(3,1)	7	(7,2,1)
Flatten layer	-	-	294	-	-	84	-	-	14
output	-	-	7	-	-	7	-	-	7

**Table 4:** Performance comparisons for detecting the existence of pairwise interactions in the AReM dataset.

	InCNN	CNN Without Interaction Filters
Accuracy	0.861	0.842
F1 score	0.859	0.838

**Table 5:** Performance comparisons with varying CNN models.

Class	InCNN +RF	InCNN +LR	CNN Without Interaction Filters	1D CNN
bending1	**0.957**	0.943	0.938	0.829
bending2	**0.909**	0.829	0.822	0.510
cycling	**0.955**	0.913	0.915	0.869
lying	0.904	**0.943**	0.930	0.915
sitting	**0.753**	0.641	0.534	0.448
standing	**0.902**	0.808	0.833	0.722
walking	**0.915**	0.905	0.918	0.876
Total Acc	**0.902**	0.855	0.842	0.740
F1 score	**0.900**	0.853	0.838	0.728

**Table 6:** Performance comparison with hand-crafted features.

Class	InCNN +RF	InCNN +LR	RF (Hand -Crafted)	LR (Hand -Crafted)
bending1	**0.957**	0.943	0.951	0.887
bending2	**0.909**	0.829	0.836	0.792
cycling	**0.955**	0.913	0.674	0.633
lying	0.904	**0.943**	**0.943**	0.916
sitting	**0.753**	0.641	0.653	0.461
standing	**0.902**	0.808	0.828	0.686
walking	**0.915**	0.905	0.748	0.689
Total Acc	**0.902**	0.855	0.803	0.721
F1 score	**0.900**	0.853	0.803	0.719

## Data Availability

The original data presented in the study are openly available in UCI Machine Learning Repository at https://archive.ics.uci.edu/dataset/366/activity+recognition+system+based+on+multisensor+data+fusion+arem (accessed on 17 August 2025), and the related study can be accessed via DOI: 10.3233/AIS-160372.
